# Enhanced Sensitivity of Patient-Derived Pediatric High-Grade Brain Tumor Xenografts to Oncolytic HSV-1 Virotherapy Correlates with Nectin-1 Expression

**DOI:** 10.1038/s41598-018-32353-x

**Published:** 2018-09-17

**Authors:** Gregory K. Friedman, Joshua D. Bernstock, Dongquan Chen, Li Nan, Blake P. Moore, Virginia M. Kelly, Samantha L. Youngblood, Catherine P. Langford, Xiaosi Han, Eric K. Ring, Elizabeth A. Beierle, G. Yancey Gillespie, James M. Markert

**Affiliations:** 1 Division of Pediatric Hematology and Oncology, Department of Pediatrics, University of Alabama at Birmingham, Birmingham, AL 35233 USA; 2 School of Medicine, University of Alabama at Birmingham, Birmingham, AL 35233 USA; 3 Division of Preventive Medicine, University of Alabama at Birmingham, Birmingham, AL 35233 USA; 4 Department of Neurosurgery, University of Alabama at Birmingham, Birmingham, AL 35233 USA; 5 Department of Neurology, University of Alabama at Birmingham, Birmingham, AL 35233 USA; 6 Department of Surgery, University of Alabama at Birmingham, Birmingham, AL 35233 USA

## Abstract

Pediatric high-grade brain tumors and adult glioblastoma are associated with significant morbidity and mortality. Oncolytic herpes simplex virus-1 (oHSV) is a promising approach to target brain tumors; oHSV G207 and M032 (encodes human interleukin-12) are currently in phase I clinical trials in children with malignant supratentorial brain tumors and adults with glioblastoma, respectively. We sought to compare the sensitivity of patient-derived pediatric malignant brain tumor and adult glioblastoma xenografts to these clinically-relevant oHSV. In so doing we found that pediatric brain tumors were more sensitive to the viruses and expressed significantly more nectin-1 (CD111) than adult glioblastoma. Pediatric embryonal and glial tumors were 74-fold and 14-fold more sensitive to M002 and 16-fold and 6-fold more sensitive to G207 than adult glioblastoma, respectively. Of note, pediatric embryonal tumors were more sensitive than glial tumors. Differences in sensitivity may be due in part to nectin-1 expression, which predicted responses to the viruses. Treatment with oHSV resulted in prolonged survival in both pediatric and adult intracranial patient-dervied tumor xenograft models. Our results suggest that pediatric brain tumors are ideal targets for oHSV and that brain tumor expression of nectin-1 may be a useful biomarker to predict patient response to oHSV.

## Introduction

Malignant brain tumors are the leading cause of cancer-related morbidity and mortality in children, and high-grade gliomas in adults likewise result in significant morbidity and mortality with a median survival of approximately 15 months for adults treated with standard therapies^1–3^. Traditional therapies for malignant brain tumors in children and adults consist of surgical resection, chemotherapy, and radiation. These therapies result in considerable neurotoxicity, especially in the developing brain of a child, and can result in long-term disabilities such as cognitive difficulties, neuroendocrine dysfunction, and neurosensory deficits in survivors^4^. Innovative therapies that are capable of selectively targeting tumor cells while sparing normal brain cells are urgently required to improve outcomes and lessen toxicities from current therapies in children and adults with brain cancer.

Engineered oncolytic HSV-1 virotherapy (oHSV) has shown great promise in targeting difficult-to-treat adult cancers as demonstrated by the recent FDA approval of the first oncolytic virus, talimogene laherparapvec (T-VEC), an oHSV that contains deletions in the neurovirulence gene γ_1_34.5 and that produces granulocyte macrophage colony-stimulating factor (GM-CSF) during replication. T-VEC improved durable response rate compared to GM-CSF alone in advanced melanoma^5^. Deletion of the γ_1_34.5 gene prevents a productive infection in normal brain cells through PKR-mediated translational arrest while maintaining the virus’ oncolytic activity against cancer cells which harbor defective signaling pathways or activating *ras* mutations that suppress cellular host antiviral responses^6–8^. After infecting tumor cells, oHSV generates progeny that are released by cytolysis and can infect adjacent tumor cells, thereby augmenting the therapeutic response. The immunogenic virus engenders an anti-tumor immune response by producing a debris field that exposes cancer cell antigens to immune effector cells for activation, and associated expression of a cytokine such as GM-CSF or interleukin-12 (IL-12) can further amplify the anti-tumor immune response. Of note, T-VEC was derived from the HSV-1 strain JS1, and in addition to the γ_1_34.5-deletetion has a deletion in ICP47 which leads to early expression of US11 and increased virus production. The γ_1_34.5-deleted/ICP47-deleted/JS1 HSV-1 caused toxicity in mice when inoculated intracerebrally at 1 × 10^5^ plaque-forming units (PFU)/mL making it undesirable for the treatment of brain tumors^9^.

Two γ_1_34.5-deleted viruses, G207, which also contains a lacZ insertion within the UL39 gene that encodes viral ribonucleotide reductase, and M032, which expresses human IL-12, are currently being studied in a phase I brain tumor clinical trial in children (clinicaltrials.gov NCT02457845) and adults (NCT02062827) respectively^10,11^. G207 was proven safe with evidence of efficacy in some patients in three completed phase I studies in adults with recurrent high-grade glioma^12–14^. However, several current shortfalls exist in the clinical advancement of oHSV to target brain tumors. There is a lack of robust preclinical studies distinguishing the ideal patient and brain tumor histopathologic types to target with oHSV, and there is no known biomarker to predict which patients are likely to respond to oHSV so that only those patients likely to respond are included on studies. Therefore, in the current study, we sought to define and compare sensitivity patterns of a panel of patient-derived xenografts including pediatric embryonal brain tumors, pediatric high-grade glial tumors, and adult glioblastoma, using clinically relevant oHSVs G207 and M002 (differs from M032 only by expressing murine IL-12 transgene instead of the human transgene) and to determine if the primary HSV-1 entry molecule nectin-1 (CD111) may be a useful biomarker to predict the response of malignant brain tumors to oHSV.

## Results

### *In vitro* cytotoxicity

**Figure 1:**
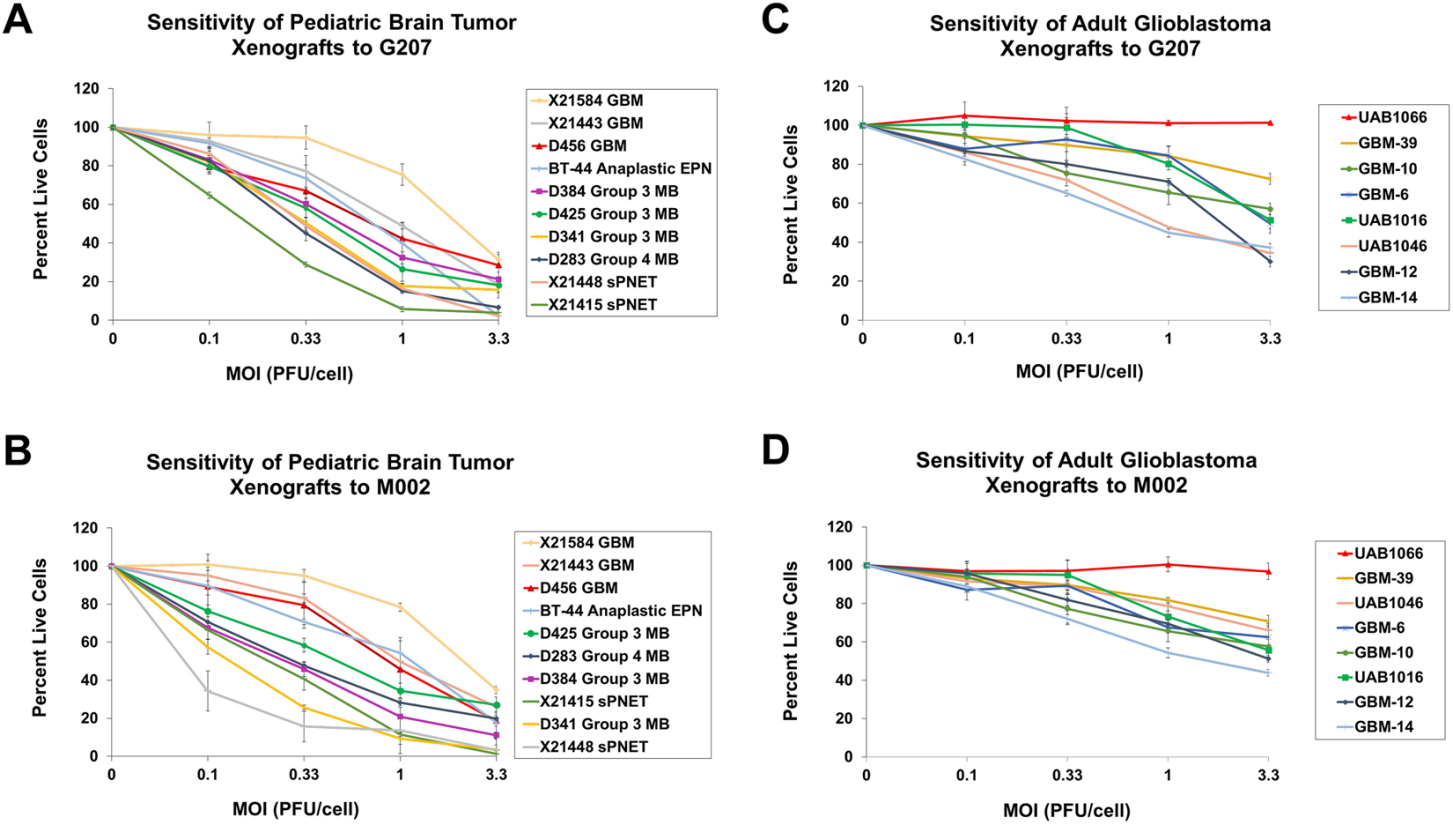
Dose-response of pediatric brain tumor and adult glioblastoma *ex vivo* cells from patient-derived xenografts to HSV G207 or M002 after 72 hours as measured by cytotoxicity assay. Plot at each dose level represents the mean of 4 replicates ± standard deviation. Sensitivity of pediatric brain tumor xenograft cells to G207 (**A**) and M002 (**B**). Sensitivity of adult glioblastoma xenograft cells to G207 (**C**) and M002 (**D**). In comparison to the pediatric tumor cells, there were significantly more adult tumor cells alive after 72 hours of G207 (p = 0.0002) and M002 (p < 0.0001) infection. MOI, multiplicity of infection; GBM, glioblastoma; EPN, ependymoma; MB, medulloblastoma; sPNET, supratentorial Primitive Neuroectodermal Tumor.

Tumor cells from 10 pediatric and 8 adult patient-derived tumors were tested for sensitivity to oHSV G207 and M002 by adding graded doses of virus ranging from 0 to 3.3 PFU/cell. The percent of cells alive compared to a control (uninfected cells) at each dose 72 hours after infection and the inhibitiory concentration required to inhibit 50% of the cells (IC_50_) were subsequently calculated for each virus. These viruses were chosen because of their clinical relevance; G207 is currently in a phase I clinical trial in children with recurrent or progressive supratentorial malignant brain tumors (NCT02457845), and M002 is identical to M032, which is currently in a phase I clinical trial in adults with recurrent malignant high-grade glioma (NCT02062827), except M002 expresses the murine IL-12 transgene and M032 expresses the human IL-12 transgene^10,11^. The pediatric tumor cells were highly sensitive to both G207 and M002; at 3.3 PFU/cell, only 2.1–31.1% (mean 14.8 ± 10.7%) (Fig. 1A) and 1.2–34.8% (mean 16.3 ± 11.3%) (Fig. 1B) of cells were alive at 72 hours after infection, respectively. The IC_50_ for the pediatric tumor cells ranged from 0.2–1.7 PFU/cell for G207 and 0.1–2.0 PFU/cell for M002. The adult tumor cells had a wider range of sensitivity to G207 and M002 at 3.3 PFU/cell with 39.1–101.2% (mean 54.1 ± 23.5%) (Fig. 1C) and 43.8–96.8% (mean 63.1 ± 16.0%) (Fig. 1D) of cells alive at 72 hours after infection, respectively. The IC_50_ for the adult tumors ranged from 1.0 to >100 PFU/cell for G207 and 1.7 to >100 PFU/cell for M002.

**Figure 2:**
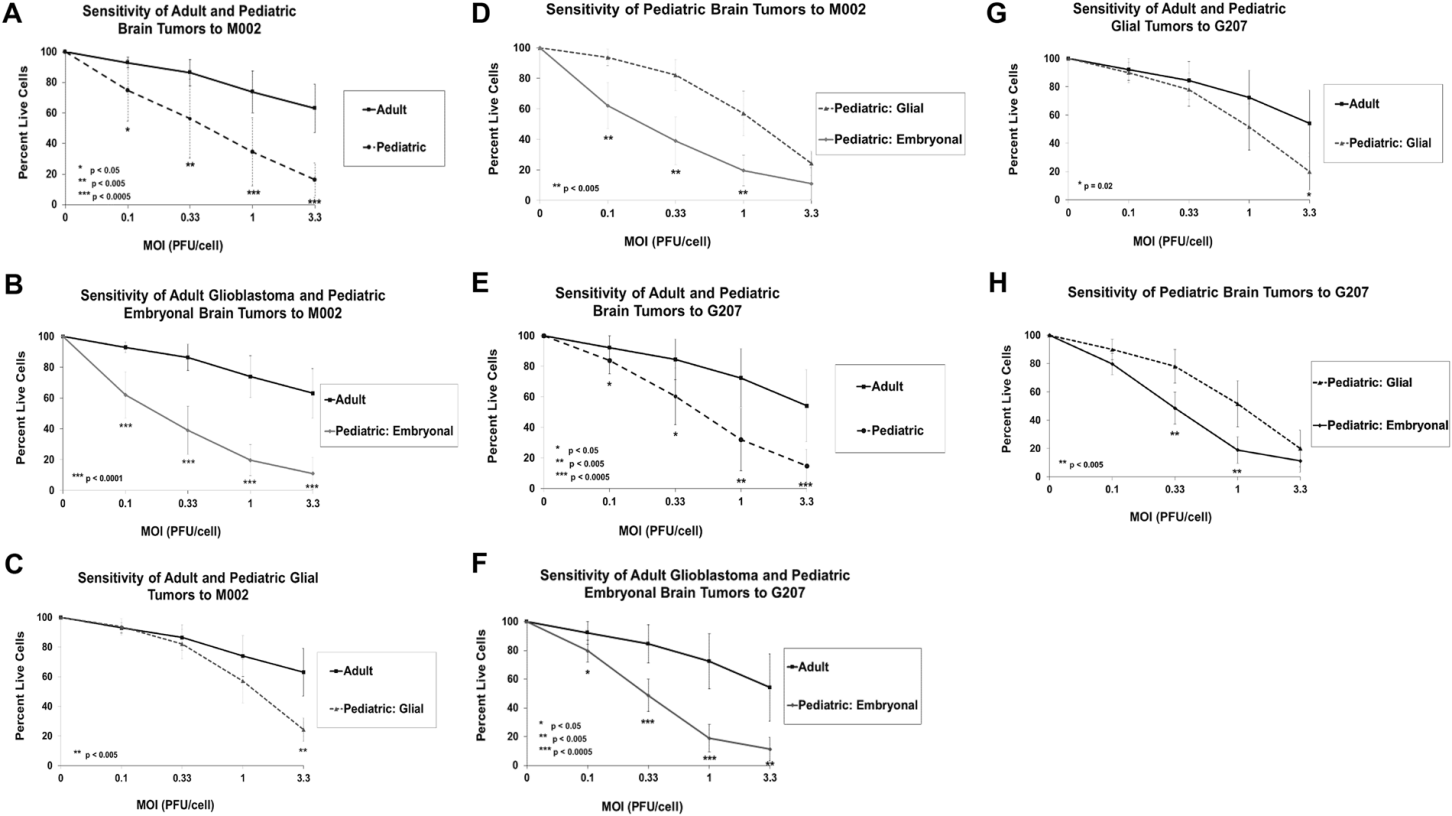
Average dose-response of pediatric brain tumor (all pediatric tumors, pediatric embryonal tumor subset, pediatric glial tumor subset) and adult glioblastoma *ex vivo* cells from patient-derived xenografts to HSV M002 and G207 after 72 hours as measured by cytotoxicity assay. Plot at each dose level represents the average ± standard deviation of each tumor group. (**A**) Comparison of adult glioblastoma tumors and all pediatric brain tumors to M002. (**B**) Comparison of adult glioblastoma tumors and pediatric embryonal tumors to M002. (**C**) Comparison of adult glioblastoma tumors and pediatric glial tumors to M002.(**D**) Comparison of pediatric glial and embryonal tumors to M002. (**E**) Comparison of adult glioblastoma tumors and all pediatric brain tumors to G207. (**F**) Comparison of adult glioblastoma tumors and pediatric embryonal tumors to G207. (**G**) Comparison of adult glioblastoma tumors and pediatric glial tumors to G207. (**H**) Comparison of pediatric glial and embryonal tumors to G207. MOI, multiplicity of infection.

We next compared the average sensitivity of the pediatric and adult brain tumors. At all dose levels (0.1, 0.33, 1.0 and 3.3 PFU/cell), the pediatric tumor cells were significantly more sensitive to M002 (Fig. 2A) and G207 (Fig. 2E) as determined by a lower percentage of live cells compared to control (uninfected cells) after 72 hours. On average the pediatric tumors were 37-fold and 11-fold more sensitive to M002 and G207, respectively. We then divided the pediatric tumors into embryonal (medulloblastoma and supratentorial neuroectodermal tumor [sPNET]) and glial (glioblastoma and ependymoma) subgroups and compared their sensitivity to the adult glial tumors and to each other. The pediatric embryonal tumor cells were 74-fold and 16-fold more sensitive to M002 (Fig. 2B) and G207 (Fig. 2F), correspondingly, compared to the adult tumor cells, whereas the pediatric glial tumor cells were 14-fold and 6-fold more sensitive to M002 (Fig. 2C) and G207 (Fig. 2G) compared to the adult tumor cells. While the pediatric embryonal tumor cells were significantly more sensitive to both viruses than the adult tumor cells at all dose levels, the pediatric glial tumor cells were significantly more sensitive than the adult tumor cells only at a dose of 3.3 PFU/cell. The pediatric embryonal tumor cells were on average 6-fold and 3-fold more sensitive to M002 (Fig. 2D) and G207 (Fig. 2H) than the pediatric glial tumor cells; however, at 3.3 PFU/cell, there was no difference in the sensitivity of the subgroups to either virus. These data indicate that pediatric brain tumors have enhanced sensitivity to oHSV M002 and G207 *in vitro*. Furthermore, pediatric embryonal tumors are the most sensitive group of tumors; however, as the dose of virus increases, pediatric glial tumors appear to be as sensitive as pediatric embryonal tumors and more sensitive than adult glial tumors.

### HSV entry molecule expression

**Figure 3:**
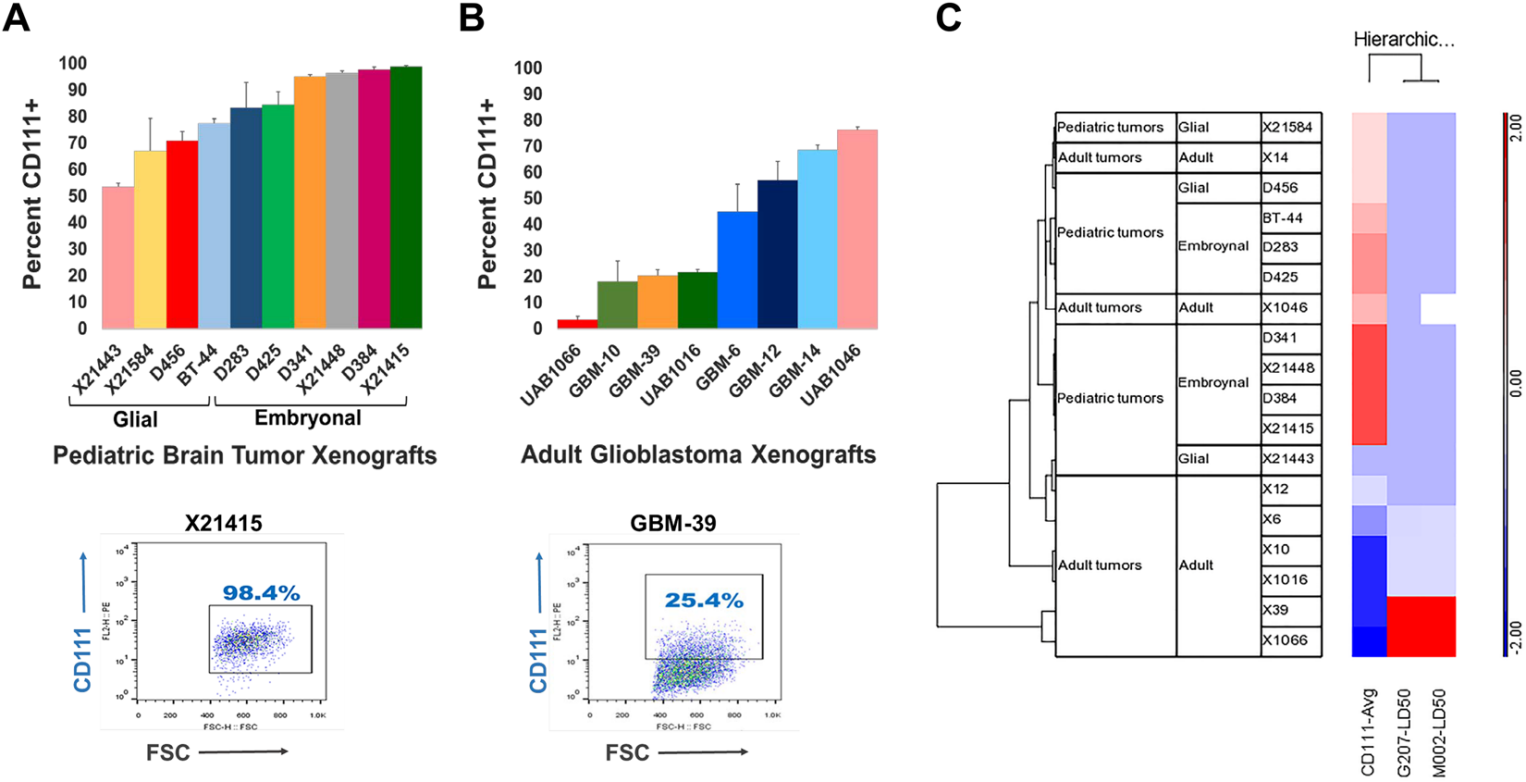
Correlation of nectin-1 (CD111) positive cells in *ex vivo* cells from patient-derived pediatric brain tumor and adult glioblastoma xenografts. (**A**) Upper panel: cells from pediatric brain tumor xenografts by glial and embryonal tumor subsets. Lower panel: example of flow cytometry of pediatric embryonal supratentorial PNET X21415. (**B**) Upper panel: cells from adult glioblastoma xenografts. Lower panel: example of flow cytometry of adult GBM-39. (**C**) Hierarchical clustering of pediatric and adult brain tumor xenografts based on nectin-1 expression and concentration required to inhibit 50% of cells (IC_50_) for G207 and M002. Color indicates greater (in red) or less (in blue) than average. A significant inverse correlation exists between nectin-1 expression and IC_50_ (M002, r = −0.73, p = 0.001; G207, r = −0.72, p = 0.001) indicating that nectin-1 percentage correlated with tumor cell sensitivity to the viruses.

We next sought to determine if there was differential expression of nectin-1 (CD111), a cell surface adhesion molecule that is the most efficient mediator of HSV entry, between the pediatric and adult tumors^15^. Freshly disaggregated cells from the panel of patient-derived pediatric high-grade brain tumors and adult glioblastoma were evaluated for expression of nectin-1 by flow cytometry. Nectin-1 expression ranged from 53.4–98.8% for the pediatric tumors (Fig. 3A) and 3.5–76.2% for the adult tumors (Fig. 3B). On average a significantly greater number of pediatric tumor cells expressed more nectin-1 than the adult tumor cells (82.4 ± 15.3% versus 38.8 ± 26.6%; p = 0.0005), suggesting that HSV-1 should be able to enter the pediatric tumor cells more efficiently than the adult tumor cells. Furthermore, the pediatric embryonal tumors expressed significantly more nectin-1 compared to the pediatric glial tumors (92.6 ± 6.9% versus 67.2 ± 10.2%; p = 0.0014) (Fig. 3A).

### Correlation between nectin-1 (CD111) expression and cytotoxicity

Since the pediatric tumor cells expressed significantly more nectin-1 than the adult tumor cells and were more sensitive than the adult tumors, we sought to determine if expression levels of nectin-1 correlated with the sensitivity of the cells to the viruses. Hierarchical clustering of the tumors based on nectin-1 and the IC_50_ values revealed a significant inverse correlation between nectin-1 expression and IC_50_ for M002 (*r* = −0.73, p = 0.001) and G207 (*r* = −0.72, p = 0.001) (Fig. 3C). These data suggest that nectin-1 expression may be a useful biomarker to predict sensitivity of brain tumors to oHSV.

### *In vivo* cytotoxicity

**Figure 4:**
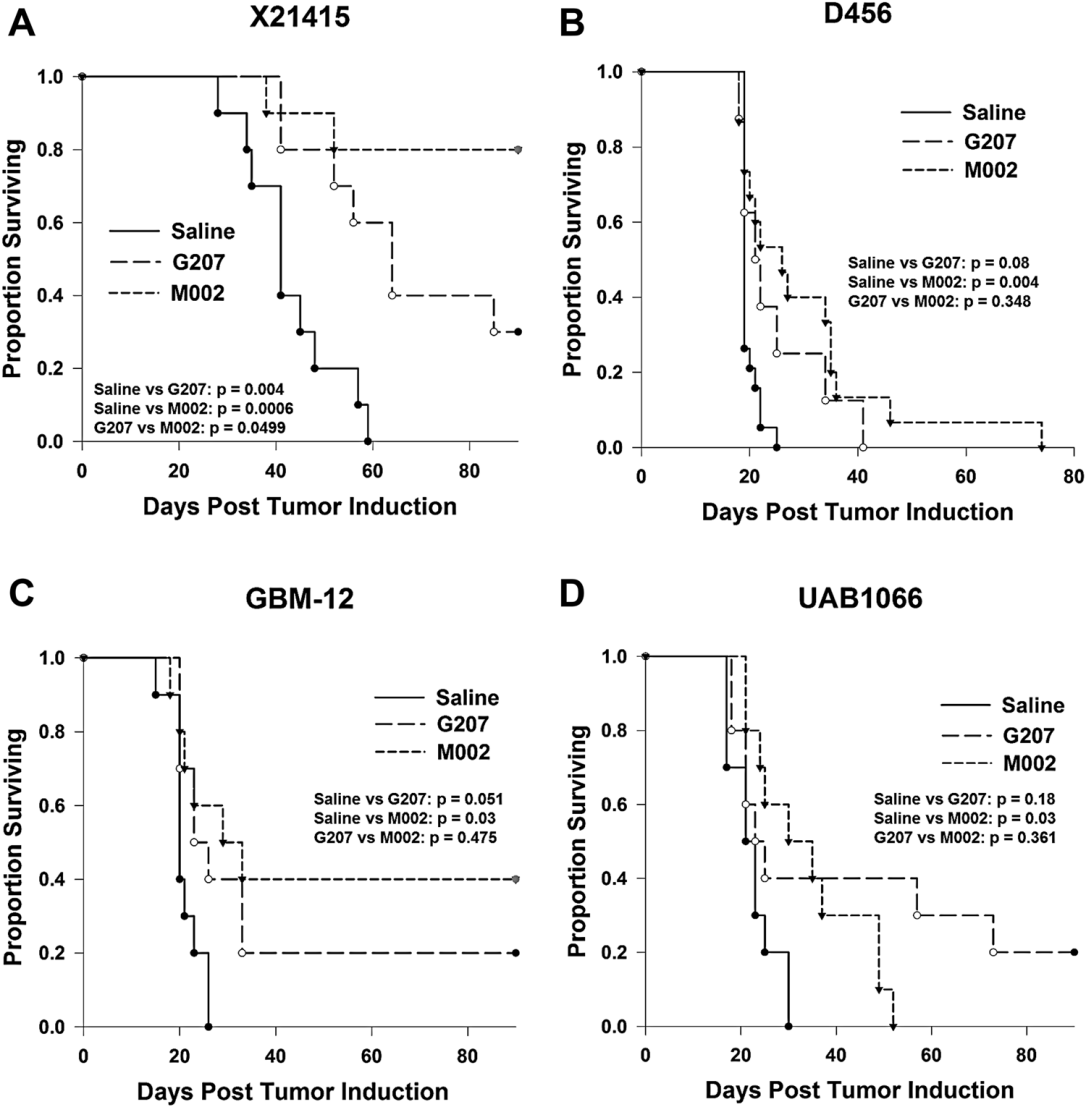
Kaplan-Meier survival plots of athymic nude mice after intracranial injection of 3–5 × 10^5^ tumors cells. Five to seven days after tumor implantation, mice received a single intratumoral injection of 5 µl of saline or 1 × 10^7^ plaque-forming units of G207 or M002. (**A**) Pediatric embryonal tumor X21415; (**B**) pediatric glioblastoma D456; (**C**) adult glioblastoma GBM-12; (**D**) adult glioblastoma UAB1066.

For *in vivo* efficacy studies, two pediatric tumors, glial tumor D456 with lower nectin-1 expression and embryonal tumor X21415 with higher nectin-1 expression, and two adult tumors, UAB1066 with lower nectin-1 expression and GBM-12 with higher nectin-1 expression were tested. Pediatric tumor X21415, which had the highest nectin-1 expression of the four tumors, was the most sensitive tumor; mean and median survival time in mice bearing intracranial tumors were significantly prolonged after treatment with a single 1 × 10^7^ PFU dose of M002 (mean 81.0 ± 8.2 days; p = 0.0006) or G207 (mean 67.3 ± 6.5 days; median 64.0 ± 6.2; p = 0.004) compared to saline (mean 42.9 ± 3.1 days; median 41.0 ± 3.1) (Fig. 4A). Eight of 10 animals in the M002 group and 3 of 10 animals in the G207 group survived to the 90-day end point of the experiment. M002 also significantly prolonged median survival in mice bearing pediatric glioblastoma D456, which had lower nectin-1 expression. Median survival time was increased from 19.0 ± 0.4 days to 30.0 ± 3.8 days (p = 0.004) after a single dose of M002. While there was trend towards increased median survival with G207 treatment (21.0 ± 2.1 days), it did not reach significance (p = 0.08) (Fig. 4B).

Similarly, M002 significantly prolonged survival in mice bearing intracranial adult GBM-12 compared to saline (29.0 ± 7.9 days versus 20.0 ± 1.5 days, p = 0.03), and G207 resulted in a trend towards prolonged survival but was not significant (23.0 ± 3.2 days, p = 0.051) (Fig. 4C). Several animals in both the M002 and G207 groups survived until the 90-day end point. UAB1066 had the lowest nectin-1 expression of the four tumors; however, this did not preclude a response to M002 with median survival increased from 21.0 ± 1.5 days to 30.0 ± 7.9 days (p = 0.03) with treatment (Fig. 4D). G207 did not significantly prolong survival in this tumor model. These data indicate that pediatric embryonal tumor X21415, with the highest nectin-1 expression, was the most sensitive of the tumor models tested *in vivo* to both M002 and G207. M002 significantly outperformed G207 in X21415, and low nectin-1 expression did not preclude a partial response.

## Discussion

Pediatric and adult high-grade brain malignancies result in high morbidity and mortality and remain a treatment challenge. Engineered oHSV has emerged as a promising approach to target highly aggressive brain tumors. We studied two viruses, G207 and M002 (expresses murine IL-12 transgene compared to M032 which expresses the human IL-12 transgene) that are currently in clinical trials in children and adults, respectively. There have been few preclinical studies exploring the ability of these viruses to target human pediatric and adult brain tumor xenografts, and no studies have compared the sensitivity between adult and pediatric tumors. Most of the prior studies have been limited by the tumor models used. Initial studies demonstrating efficacy of G207 and M002 utilized established glioblastoma cell lines maintained in tissue culture as monolayers with Dulbecco’s Modified Eagle’s Medium and 10% fetal bovine serum^16,17^. These culture conditions can result in the loss of tumor heterogeneity as well as genotypic and phenotypic changes over time that result in tumor cells that are dissimilar from the original tumor. All of the tumors in the current study were maintained as xenografts. For *in vitro* studies, we used short term culture with cells grown as neurospheres in stem cell-defined medium designed to minimize cell differentiation.

We demonstrated that in the culture conditions described above, the patient-derived pediatric brain tumor xenografts express nectin-1 on the cell surface in significantly greater amounts than the adult glioblastoma xenografts. Nectin-1 is a cell-cell adhesion molecule found on variety of normal and malignant cells and is involved in the organization of adherens and tight junctions^15^. We chose to focus our studies on nectin-1 as opposed to other known entry molecules such as herpes virus entry mediator (HVEM) or nectin-2, because nectin-1 is more efficient at promoting HSV entry, HVEM is not expressed in neuroglial cells to a significant degree, and nectin-2 has been shown to be inactive for HSV-1 entry^15,18,19^. Interestingly, despite being an adhesion molecule, nectin-1 expression is associated with metastatic disease in human breast cancer and in highly migratory and invasive squamous cell carcinoma^20,21^. Pediatric brain tumors have a greater propensity to metastasize compared to adult glioblastoma, and therefore, we surmise this may be related to the finding that nectin-1 is found in greater amounts on the pediatric tumors. Supporting this supposition is the increased nectin-1 expression seen in pediatric embryonal tumors, which have an even greater likelihood of metastasizing than pediatric glial tumors.

Importantly, nectin-1 expression correlated with the sensitivity of the brain tumor xenografts to G207 and M002. Nectin-1 facilitates efficient HSV-1 entry; HSV-1 glycoprotein D binds to nectin-1 resulting in the fusion of the virus envelope with the cell membrane enabling the virus to enter the cell^22^; nectin-1 is ultimately internalized within infected cells^23,24^. We previously showed that a human glioblastoma xenograft with <20% nectin-1 expression was less sensitive to infection and killing by HSV-1 than tumors with higher expression, and other groups determined that nectin-1 predicted oncolysis by HSV-1 in squamous cell carcinoma, thyroid cancer, and EBV-associated lymphoproliferative disease^25–28^. Of note, in preclinical animal models and in human clinical trials, subsequent doses of virus were effective at controlling tumor indicating that uninfected tumor cells maintain sensitivity to oHSV^14,29^. While nectin-1 predicts sensitivity in some tumor types, expression levels do not correlate with oHSV sensitivity in all tumor types; sensitivity of neuroblastoma cell lines to γ_1_34.5-deleted HSV1716 was independent of nectin-1 or other known entry molecules such as nectin-2, herpes virus entry mediator (HVEM), or 3-OS heparin sulfate^30^. The varying levels of sensitivity seen in the neuroblastoma lines was attributed to cellular anti-viral response and not virus entry. Furthermore, there was no correlation between nectin-1 levels and G207 viral yields in malignant peripheral nerve sheath tumor (MPNST) cell lines, and overexpression of nectin-1 in resistant MPNST cell lines did not improve viral yields or confer a sensitive phenotype to the resistant cells^31^.

Our finding that nectin-1 expression correlates with sensitivity to oHSV in malignant brain tumor xenografts has significance for current and future oHSV brain tumor trials, as it may represent an important biomarker to predict patient response to treatment with oHSV. The heterogeneity of nectin-1 expression seen between different brain tumor histopathological types and among tumors of the same histology in this study and other studies also support the possible role of nectin-1 as a biomarker for oHSV studies^32,33^. Tumor expression levels may be useful as inclusion/exclusion criteria to ensure that patients most likely to benefit from the therapy are included on studies and that those unlikely to benefit are excluded. This will need to be validated in clinical trials to determine if there is a specific threshold of nectin-1 required for a response and highlights the importance of obtaining and evaluating patient tumor tissue. Of note, while cell entry is critical for oHSV infection, other factors which may affect replication such as activated Ras, p38 MAPK, or interferon cell signaling pathways may also play a role and require additional investigation^34,35^.

Corresponding with the significant differences in nectin-1 expression between pediatric and adult tumors, the pediatric tumors were significantly more sensitive, by a 1.0–1.6 log difference in dose, to G207 and M002 than the adult tumors. This suggests that pediatric brain tumors may be ideal targets for oHSV virotherapy. While pediatric glial tumors were more sensitive to the viruses than the adult glial tumors, pediatric embryonal tumors were the most sensitive brain tumor type. We previously showed that the pediatric embryonal tumor type, medulloblastoma, including molecular group 3 tumors which portend a poor prognosis, and the chemo- and radio-resistant medulloblastoma stem cells, are highly sensitive to killing by oHSV *in vitro* and *in vivo*^36^. In the current study we have demonstrated that sPNET, an embryonal tumor, is likewise highly sensitive to killing by a single dose of oHSV G207 and M002, which resulted in 80% of the animals surviving to the endpoint of the experiment. Additionally, Studebaker *et al*. found that a single dose of oHSV significantly prolonged survival in mice bearing intracranial medulloblastoma and atypical teratoid/rhabdoid tumor (AT/RT), another tumor of embryonal origin^37^. Together, these studies indicate that pediatric embryonal tumors may be the ideal tumor type for oHSV virotherapy. Nevertheless, pediatric glial tumors were sensitive in the current study, and in a recent study, oHSV inhibited invasion and migration in three pediatric high-grade glioma cell lines including one diffuse intrinsic pontine glioma line suggesting that pediatric high-grade gliomas are an attractive target for oHSV^38^. In addition to the enhanced sensitivity of pediatric brain tumors to oHSV, the lack of pre-existing immunity to HSV-1 in most children may enable the virus to persist for longer which may enhance the oncolytic effect of the virus and lead to a more robust anti-tumor immune response^39^; this is being explored in the current G207 pediatric clinical trial^10^.

While the adult glioblastomas were not as sensitive as the pediatric tumors, we still saw significant cell killing in all but one of the tumors *in vitro*, and a single dose of M002 prolonged survival in mice bearing that tumor model as well as another adult glioblastoma xenograft. These findings indicate that adult glioblastoma are sensitive to killing by M002, and not all *in vivo* anti-tumor responses can be predicted based on the *in vitro* response. A significant *in vivo* response may occur even in situations where the tumor appears resistant *in vitro*; Leddon, *et al*. demonstrated that an antitumor immune response may occur in the absence of virus permissivity in murine sarcomas^40^. Based on the fact that M002 prolonged survival in that tumor model but G207 did not, we speculate that the proinflammatory cytokine IL-12 produced by M002 may have had an antitumor effect. IL-12 has been shown to mediate type 1 helper T cell (Th1) responses, activate natural killer (NK) cells, and contain antiangiogenic and antitumor properties^41,42^. The precise mechanism for the *in vivo* response of the adult glioblastoma xenograft that appeared resistant *in vitro* requires further investigation. A limitation of our study is the lack of a fully intact immune system in the athymic nude mouse model used for the experiments, which was necessitated by the study of human tumors. Since both G207 and M002 can elicit antitumor immunity that is associated with a cytotoxic T cell response, the athymic mouse model may actually underestimate the sensitivity of the tumors due to the loss of the antitumor immune response that may have occurred in an immunocompetent animal. The lack of available murine tumor models prevents a parallel study in immunocompetent mice that would fully translate to the human tumors we studied.

Our study provides a detailed examination of the general sensitivity of both pediatric embryonal and glial tumors and adult glioblastoma to oHSV and provides insight into the potential efficacy of G207 and M032, two HSVs that have been used in North American clinical brain tumor trials. G207 was safely used in three phase I studies in 36 adults with recurrent high-grade gliomas. The virus was safe without a maximum tolerated dose reached when inoculated up to 3 × 10^9^ PFU in five sites intratumorally, when given in two doses (one administered pre-tumor resection and one post resection in the resection cavity) totaling 1.15 × 10^9^ PFU, and when given by stereotactic intratumoral administration at 1 × 10^9^ PFU followed within 24 hours by a single 5 Gy dose of radiation^12–14^. While the studies were only designed to demonstrate safety, nearly half of the adult patients demonstrated radiographic and/or neuropathologic evidence of an anti-tumor response. The safety and tolerability of intratumoral G207 alone and combined with radiation is currently being studied in a first of its kind phase I clinical trial in children age 3–18 with recurrent or progressive supratentorial malignant brain tumors (NCT02457845)^10^. Our preclinical data suggest that responses in this study may be even better than the adult clinical trials. M032, a second-generation virus that expresses human IL-12 (the clinical version of M002), is the first oHSV expressing a human foreign gene to be studied in the human brain. The phase I clinical trial (NCT02062827) in adults with recurrent high-grade gliomas in currently accruing patients, and our preclinical data suggest that M032 may outperform G207^11^. Taken together, our data indicate that additional oHSV trials in children and adults are strongly warranted.

## Materials and Methods

### Patient-derived brain tumor xenografts and mice

Animal experiments were approved by the University of Alabama at Birmingham (UAB) Institutional Animal Care and Use Committee (IACUC) (APN130409874 and APN 130509395) and were performed in accordance with all relevant experimental guidelines. Further, all experiments utilizing human xenografts (obtained after informed consent was acquired from either the patient and/or parent(s)/legal guardian) were performed in accordance with policies set forth by the UAB Institutional Review Board (IRB) in accordance with National Institutes of Health (NIH) guidelines; approved UAB protocol (X050415007).

Ten pediatric brain tumor xenografts were used for the studies: 4 glial tumors (glioblastomas D456, X21443, X21584 and anaplastic ependymoma BT-44) and 6 embryonal tumors (medulloblastoma D283, D341, D384, D425 and sPNET X21415 and X21448). D456, D283, D341, D384, and D425 were established from pediatric patients and were provided by Darell D. Bigner, M.D., Ph.D., Duke University Medical Center^43–45^. D341, D384, and D425 are molecular group 3 tumors with *MYC* amplification, and D283 is a group 4 tumor^36,46,47^. BT-44 was established by the Pediatric Preclinical Testing Program and provided by Peter Houghton, Ph.D., UT Health San Antonio^48^. X21443 (obtained from a 17-year-old African American female patient), X21584 (12-year-old Caucasian male), X21415 (8-year-old African American female) and X21448 (11-month-old Caucasian female) were established as xenografts initially by direct implantation of freshly resected patient tumors samples at Children’s of Alabama into the flanks of immunocompromised athymic nude (nu/nu) mice. GBM-6, GBM-10, GBM −12, UAB 1016, UAB1046, and UAB1066 have been previously described and were also established by direct implantation of freshly resected human glioblastoma tissue into the flanks of athymic mice^25^. GBM-6, GBM-10, GBM −12, GBM-14, and GBM-39 were provided by C. David James, Ph.D., and Jann Sarkaria, M.D., Mayo Clinic. Tumor histopathology was defined and confirmed by neuropathologists at Children’s of Alabama and the University of Alabama at Birmingham (UAB). Authentication of all human cell lines was determined by Short Tandem Repeat (STR) profiling performed by the UAB Heflin Center for Genomic Science. The xenografts were maintained by serial transplantation in athymic nude mice.

### Genetically engineered herpes simplex viruses

G207 and M002 have been previously described^16,17^. Briefly, G207 and M002 contain deletion of both copies of the γ_1_34.5 gene. G207 also contains a lacZ insertion into the UL39 gene which encodes viral ribonucleotide reductase, and M002 expresses murine IL-12 constitutively under the transcriptional control of the murine *EGR-1* promoter. In previous studies, M002 has been shown to produce physiologically relevant amounts of IL-12^17,49^. The UL39 gene is intact in M002 which may explain the replication advantage seen in preclinical studies when compared to G207 in long-term established glioma cell lines^17^.

### Tumor disaggregation and tissue culture

Tumors were aseptically harvested from the flanks of nude mice and disaggregated into single cell suspensions using a gentleMacs Dissociator® (Miltenyi Biotec, Auburn, CA) per manufacturer’s standard protocol instructions as previously described^50^. Briefly, after the cells were washed twice with RPMI1640 (ATCC, Manassas, VA) (200xG, 8 minutes, room temp), the pellets were suspended in NeuroBasal medium (Invitrogen, Grand Island, NY) with fibroblast growth factor-β (Invitrogen) and epidermal growth factor (Invitrogen) at 10 ng/ml, 2% B-27 supplement without vitamin A (Invitrogen), 2 mM L-glutamine, amphotericin B (250 µg/ml) and gentamicin (50 µg/ml). Medium was exchanged as needed.

### *In vitro* cytotoxicity assay

Cells were dissociated with Accutase and plated at 10^4^ cells/well in 96 well plates. After overnight culture, graded doses from 0 to 3.3 plaque-forming units (PFU) per cell of G207 or M002 were added to each row and cytotoxicity was measured 72 hours post-infection with alamarBlue® (Life Technologies, Grand Island, NY) as previously described^23,25,50^. Graded doses of the virus were internally compared to mock-treated controls, which were included with each experiment and represented 100% tumor cell survival. Color changes of alamarBlue® were quantified with a BioTek microplate spectrophotometer (Winooski, VT), and OD595-562 nm values were used to calculate the IC_50_. (N ≥ 4).

### Flow cytometry analysis

Flow cytometry was performed as previously described^22,26,36^. Briefly, cells were dissociated into a single cell suspension using Accutase (Innovative Cell Technologies, San Diego, CA). A phycoerytherin-conjugated anti-human CD111 (clone R1.302; Biolegend, San Diego, CA) and a mouse IgG1 isotype control antibody (Miltenyi Biotec) were used. Analysis was performed on a BD LSR II Flow Cytometer or FACSCaliber (BD Biosciences, San Jose, CA) by the UAB Flow Cytometry Core Facility. The side-scatter versus forward-scatter light profiles of a control sample of cells were used to set control gates for each individual xenograft, and the results were expressed as a percentage of gated cells based on antibody binding using FlowJo v10 software (Tree Star Inc., Ashland, OR). Mean values were calculated from multiple determinations on separate dates and with separate cultures (N ≥ 3).

### *In vivo* survival study

Nude mice were stereotactically injected with 2.5–5 × 10^5^ tumor cells in 5 µL intracranially as previously described^36^. After 5–7 days, mice, in random cohorts of 10 each, received 1 × 10^7^ PFU of virus (G207 or M002) or saline stereotactically inoculated at the same site of tumor inoculation. Mice were assessed daily for toxicity, moribund mice were euthanized, and the date of death was recorded. Experiments were carried for 90 days.

### Statistical analyses

The correlation coefficient of IC_50_ of either virus to the average expression of nectin-1 was calculated using Pearson linear methods. The hierarchical clustering was performed after z-normalization of these values to average of 0 and SD of 1 using Partek Genomics Suite (Partek Incorporated, St. Louis, MO). Student’s t-test analyses for significance of mean differences were performed using Microsoft Excel (Microsoft Corp, Redmond, WA). Survival curves were created with SigmaPlot version 12.0 (Systat Software, Inc., San Jose, CA) using Kaplan–Meier analysis and median survival time. Survival between groups was compared with the log-rank test. A p value of ≤ 0.05 was considered significant.
